# A Method of Generating Real-Time Natural Light Color Temperature Cycle for Circadian Lighting Service

**DOI:** 10.3390/s23020883

**Published:** 2023-01-12

**Authors:** Seung-Taek Oh, Deog-Hyeon Ga, Jae-Hyun Lim

**Affiliations:** 1 Smart Natural Space Research Center, Kongju National University, Cheonan 31080, Republic of Korea; 2 Department of Computer Science & Engineering, Kongju National University, Cheonan 31080, Republic of Korea; 3 Department of Urban Systems Engineering, Kongju National University, Cheonan 31080, Republic of Korea

**Keywords:** circadian lighting, lighting service, natural light, color temperature, cycle

## Abstract

The light intensity and color temperature of natural light periodically change and promote the circadian entrainment of the human body. In addition, the color temperature cycle of natural light that is unique to each region is formed by its location and geographic and environmental factors, affecting the health of its residents. Research on lighting and construction to provide the color temperature of real-time natural light has continued to provide the beneficial effect of natural indoor lighting. However, lighting technology that provides the real-time color temperature of natural light could not be realized since it is challenging to select a color temperature cycle zone due to abrupt color temperature changes at sunrise and sunset. Such drastic shifts cause an irregular measurement of color temperature over time due to general weather or atmospheric conditions. In a previous study, a method of generating a color temperature cycle using deep learning was introduced, but the performance at the beginning and end of the color temperature cycle was unreliable. Therefore, this study proposes generating a real-time natural light color temperature cycle for the circadian lighting service. The characteristics of the daily color temperature cycle were analyzed based on the measured natural light characteristics database, and a data set for learning was established. To improve the color temperature cycle generation performance, a deep learning (TadGAN) model was implemented by searching for the lowest point of the color temperature at the start and end points of the color temperature cycle and applying the boot and ending datasets to these points. The color temperature cycle zone was accurately detected in real-time in the experiment, and the generation performance of the color temperature cycle was maintained at the beginning and end of the color temperature cycle. The mean absolute error decreased by about 67%, confirming the generation of a more accurate real-time color temperature cycle.

## 1. Introduction

When realizing lighting technology for people, the characteristics of natural light are an essential reference factor [1,2]. Because circadian rhythm, which influences overall human physical activity, is controlled by the spectrum, duration, intensity, and timing of light, natural light is the ideal factor for promoting circadian entrainment and health [3,4]. The characteristics of natural light are beneficial to human health, but they continuously change over a cycle of days or years [5]. Recently, research has been conducted on a lighting technology that reproduced the color temperature cycle of natural light, which helped to maintain biological rhythms by delivering the benefits of natural light to the room [6]. Beyond the early technology that provided illuminance and color temperature that changed with time, another technology evolved to reproduce the characteristics of natural light more realistically [7]. A technology that can be applied to natural light simulation was introduced by developing a white LED capable of adjusting the color temperature (CCT) from 3615 K to 15,177 K through a “Phosphor Pattern” and a “Thermal-Modulating Optical Film” [8]. Lightings that emulated the cycle of natural light on a daily or yearly basis and provided the color temperature of natural light, which differed for each region using regional information of latitude and longitude, were released [9,10]. However, most of these technologies provided a color temperature cycle derived through calculations on sunny days only or a color temperature analysis of measured natural light. Natural light changes irregularly due to fluctuations in the daily or yearly cycle formed by the Earth’s rotation and revolution, as well as the atmospheric conditions or weather created when sunlight reaches the ground [11]. The analysis result of the measured data of natural light in Korea revealed that the color temperature cycle having a constant parabolic pattern was observed only on clear days about 20% of the year, and a color temperature cycle with a mixed irregular pattern was present on most days [12]. In addition, the data on the amount of sunlight and precipitation in major countries in the world show that the color temperature cycle on most days is irregular. It is expected that a unique color temperature cycle will be formed for each region due to the location of latitude and longitude and the influence of geographical characteristics such as coasts and islands [13,14,15]. For a long time, the color temperature cycle uniquely formed in each region impacted the health and physical activity of the residents of that region. Therefore, the ideal way to reproduce the color temperature of natural light is to provide the measured color temperature in real-time in the area, regardless of whether the color temperature distribution is irregular. However, it is difficult to realize a lighting service using the measured color temperature of natural light since there are numerous cases where the color temperature change is relatively different, and related studies are scarce [16].

To reproduce the color temperature cycle of natural light in real-time, the measured color temperature values by the hour between sunrise and sunset should be provided. However, it was not easy to provide a color temperature cycle based only on the time information of sunrise and sunset provided by an astronomical or meteorological institution; the color temperature difference around sunrise and sunset is sometimes more than 3000 K [17]. It was also challenging to determine the start and end points of the natural light color temperature cycle, especially for cloudy days or unstable atmospheric conditions. Although a specific change pattern could be observed in the sunlight generated by the Earth’s rotation and revolution, a low color temperature was formed throughout the day due to the influence of cloudy weather on the ground, so periodic characteristics may not be observed [16]. Even when a color temperature with a wide range of change was measured due to the influence of clouds or rain showers, a pattern of irregular color temperature cycles arose, making it impossible to create a real-time color temperature cycle for lighting service. In a previous study, a constant parabolic color temperature cycle was generated on a sunny day using a formula, but the periodic characteristics of the color temperature distribution mixed with various irregular change patterns could not be derived. If an unsupervised learning method such as TadGAN was applied, a cycle pattern similar to the original could be imitated, and the cycle could be generated by inputting the color temperature data at a particular time interval [18,19]. However, due to the characteristics of TadGAN, which was only performed after a time series sequence of a specific window size was configured, the color temperature cycle was not generated, or a delay time occurred at the beginning and end of the color temperature cycle, which had the most significant effect on the human body, such as sleep and wakefulness. Therefore, the lighting service that provided real-time color temperature could not be realized [20].

Therefore, this study proposes a method of generating a real-time natural light color temperature cycle to realize the circadian lighting service that provides the room’s color temperature of external natural light. First, the zone and distribution characteristics of the daily color temperature cycle were analyzed using the natural light characteristic database collected through measurement. Next, a data set for learning the natural light color temperature cycle pattern was built, and a deep learning model (TadGAN) was set to generate the color temperature cycle. After that, the color temperature cycle was specified by judging whether the color temperature cycle started and ended based on the input color temperature measured in real-time. The boot and ending data sets were created and applied to improve the color temperature cycle generation performance at the target time. A deep learning model (TadGAN) was then applied to generate a real-time color temperature cycle. Through experiments, it was found that the lighting technology that reproduced the real-time color temperature cycle for various and irregular color temperature cycles with time could be realized.

## 2. Analysis of Measured Natural Light Characteristics and Construction of Data Set

**Figure 1:**
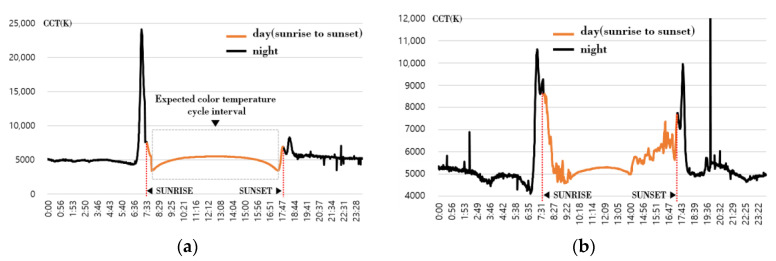
Color temperature cycle from sunrise to sunset. (**a**) Example of precise color temperature distribution (31 December 2020), (**b**) Example of unclear color temperature distribution (29 January 2021).

The natural light characteristic database collected through measurement was used to analyze the natural light color temperature cycle characteristics. The characteristics of natural light were measured by applying a spectroradiometer (CAS 140CT, Instruments, Germany) and solar tracker equipment at our university, located at latitude 36.850 and longitude 127.149. The spectroradiometer was installed in a housing with a constant temperature function to enable outdoor measurement considering the domestic weather environment. A cover made of quartz glass material (Quartz Disk, Hanjin Quartz, Republic of Korea) was applied to the light receiving part to enable measurement even in the rain [21,22]. Natural light was measured every 1 min, and optical characteristics such as spectral irradiance, illuminance, and color temperature were collected and calculated to build a database of optical characteristics. In this study, daily color temperature data for more than one year (from June 2020 to July 2021) were extracted to confirm the characteristics of the color temperature cycle. Excluding days where measurement was difficult due to harsh weather, 298 days of color temperature data were adopted to analyze patterns of color temperature cycles for various days. Figure 1 shows examples of the acquisition result of the daily color temperature cycle.

**Figure 2:**
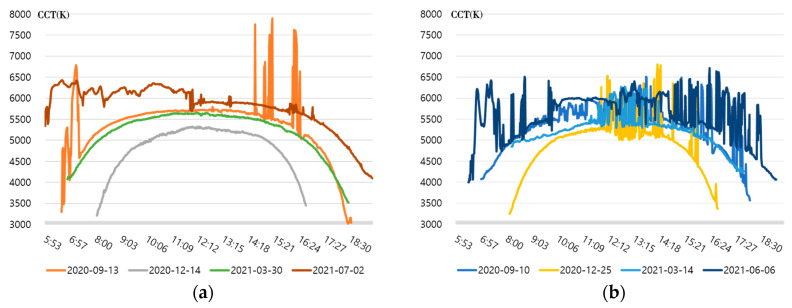
Color temperature distribution by hour according to irregular color temperature inclusion. (**a**) Irregular color temperature inclusion of less than 15%, (**b**) Irregular color temperature inclusion exceeding 15%.

In Figure 1, the dark (black) part is the color temperature at night when there is no sun, and the part displayed with bright color inside the dotted sunrise and sunset is the expected color temperature cycle zone. Figure 1a shows a parabolic distribution pattern with a transparent color temperature cycle in the inner zone after sunrise and before sunset due to the color temperature change on a clear day. Days showing the same pattern as Figure 1a were rare in all measurement days. On most days, irregular color temperature distribution was mixed in part or whole, as shown in Figure 1b. In both Figure 1a,b, zones in which the color temperature changed rapidly around sunrise and sunset were observed, and for the daily color temperature cycle, it seemed reasonable to extract the color temperature change over time after searching for the lowest point of the color temperature at the corresponding time. However, as shown in Figure 1b, it was difficult to select the lowest point of color temperature after sunrise and before sunset on most days, and it would be difficult to create a real-time natural light color temperature cycle if there was an irregular color temperature pattern in the zone. Based on the analysis result in Figure 1, the color temperature cycle between the lowest points of the color temperatures at sunrise and sunset was extracted. The regular or irregular color temperature cycle, one day each by season, was selected and displayed in Figure 2. Since there was no separate standard for indicating the regularity of the color temperature cycle, the case where the color temperature difference at 1-min intervals was 50 K or more was classified as an irregular color temperature. In the color temperature cycle, days with an irregular color temperature inclusion ratio of 15% or less and days exceeding 15% were selected and presented in Figure 2.

As shown in Figure 2a, the days with an irregular color temperature inclusion ratio of 15% or less showed an overall uniform color temperature cycle or included irregular color temperature zones with brief time intervals. On one particular day (12 January 2021), the overall color temperature was 5500 K or higher from around sunrise, and a parabolic color temperature cycle was not formed because the weather was generally cloudy from the morning. Figure 2b shows the days when the ratio of irregular color temperature exceeded 15%, where the hourly color temperature was highly irregular, and the color temperature cycle in the form of a parabola was short or difficult to identify with the naked eye; therefore, it did not seem easy to draw a color temperature cycle. Table 1 shows the analysis results according to the ratio of color temperature anomalies for all measurement days, including the days in Figure 2.

**Figure 3:**
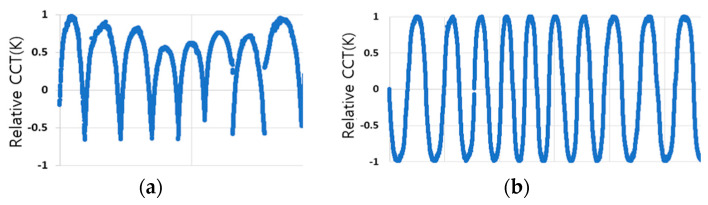
Learning dataset. (**a**) Initial learning dataset. (**b**) After applying inverse parabola padding.

As shown in Table 1, there were only 71 days (23.8%) with 0 to 5% of irregular color temperatures where the ideal color temperature cycle in the form of a parabola was observed among 298 days of measurement. The days with an irregular color temperature inclusion ratio of 5 to 10%, which rendered it easy to generate a color temperature cycle, were 47 days, while the days with 10 to 15% of irregular color temperature inclusion were 30 days. In addition, it was confirmed that on 97% of the measured days, the ratio of irregular color temperature included was within 50%. If creating a color temperature cycle of natural light that is different every day and difficult to predict through a deep learning model is desirable, it is necessary to learn about various actual light color temperature cycle patterns. To learn the deep learning model for generating the natural light color temperature cycle, this study determined the color temperature values for each hour and extracted them for 148 days (49.7%) with an anomaly inclusion rate of 15% or less. After that, generating the color temperature cycles for the measured color temperatures in real-time was attempted by connecting them to construct a dataset and executing the deep learning model. Figure 3 shows the learning data set that connected the color temperature cycle of the day when the irregular color temperature inclusion ratio was within 15%.

The figure shows the initial learning data set connected after extracting the measured color temperature according to the change of time per day and normalizing it to a relative value of +1 to −1. The color temperature cycle was too low in some cases due to the prominent irregular color temperature. In this case, the irregular color temperature was excluded. Despite this process, the previous day’s sunset and the latter’s sunrise were attached, resulting in an artificial form in Figure 3a, in which the color temperature cycle on both days changed sharply. When the time series data of such a pattern is applied to a deep learning model, sharp points may be crushed or not well-expressed. In the lighting that reproduces natural light, the start and end of the color temperature cycle that affects human health, such as sleep and wakefulness, that is, the color temperature around sunrise and sunset, is essential. Therefore, pre-processing is required to improve the generation performance of the color temperature cycle of the affected point. The first and second parts of the parabola-shaped daily color temperature cycle were divided from the highest color temperature value. Consequently, each part was symmetrically inverted (inverse parabolic padding) and connected so that the daily color temperature cycle was smoothly connected, as shown in Figure 3b. A learning dataset that stabilized the sudden color temperature change at sunrise and sunset points was constructed through this process.

## 3. A Method of Generating a Real-Time Natural Light Color Temperature Cycle

**Figure 4:**
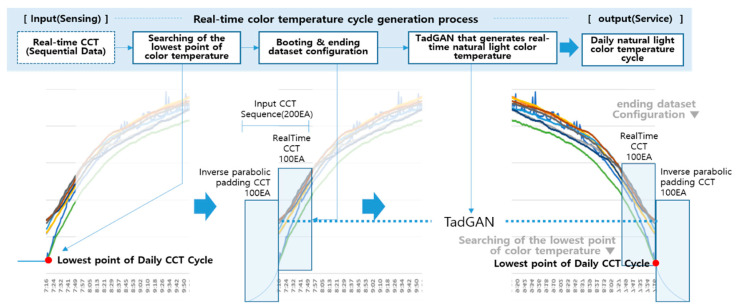
A process of generating real-time natural light color temperature cycle.

For the realization of natural light lighting service, this study proposed a method of generating a color temperature cycle in real-time for the color temperature of natural light based on time, which showed a diverse and irregular change pattern. In the proposed method, when the natural light color temperature was measured and input at each location, the lowest point of the color temperature was searched to check whether it was included in the daily color temperature cycle zone. After that, the boot and ending datasets were constructed using the time series data of the natural light color temperature continuously input by time. Subsequently, a deep learning model (TadGAN) learned through the measured natural light color temperature data set was applied to generate a real-time natural light color temperature cycle. The primary process of the proposed method is shown in Figure 4.

First, in the input stage, the color temperature of natural light was measured in real-time through an optical characteristic measuring device. A high-performance optical characteristic measuring device, such as a spectroradiometer or a slight color temperature device based on an RGB sensor, could be applied in this case. The color temperature was constantly measured at certain intervals, and these data were input. This study adopted a spectroradiometer (CAS 140CT, Instrument, Munich, Germany). Since the learning dataset extracted in Section 2 was the color temperature data measured at 1-min intervals, the color temperature of real-time natural light was also measured and input at 1-min intervals. When the measured color temperature of natural light was input in real-time, it was often difficult to determine the start time of the daily color temperature cycle, as shown in Figure 3 in Section 2. Therefore, a search algorithm for the lowest point of the color temperature was implemented and applied to determine whether the input color temperature was included in the daily color temperature cycle. A search algorithm for the lowest point of the color temperature determined whether the real-time input color temperature was the start and end point of the color temperature cycle. Table 2 shows the process of searching the color temperature cycle, that is, the lowest point of color temperature at sunrise and sunset, and setting the color temperature cycle interval.

First, in STEP 0, information on the sunrise and sunset times of the day provided by an astronomical or meteorological institution (Korea Astronomy Research Institute) was received. In the analysis results of the characteristics of natural light in Section 2, the daily natural light color temperature cycle started after elapsing at a specific time after sunrise and ended before a particular time of sunset was considered. The search for the lowest point of color temperature started at sunrise, officially provided by the national institution. In STEP 1, whether the current color temperature corresponded to the expected monthly minimum was compared. At this time, the expected monthly lowest was derived by analyzing the raw light characteristic data in Section 2. The expected lowest point occurrence zone was determined by classifying the natural light characteristics by month and calculating the ratio of the time when the lowest point occurred for each month. The results are shown in Table 3.

As shown in Table 3, the lowest point of color temperature was observed within a maximum of 75 min (relative time 12.67%) after sunrise in January and 67 min (relative time 11.67%) in December. The time at which the lowest point of color temperature was observed for each month was different. Based on the analysis results in Table 3, whether the measured time point corresponded to the monthly expected lowest point zone according to the monthly information on the measurement date was assessed. If it fell under the expected lowest point occurrence zone, STEP 2 was performed to see whether the currently input color temperature fell under the measured color temperature zone at the beginning and end of the color temperature. For this purpose, the monthly distribution of the measured lowest color temperature observed at the time of occurrence of the lowest point was analyzed. The analysis results based on the natural light characteristic data in Section 2 showed that the lowest color temperature in the zone of the lowest color temperature where the color temperature cycle started was in a range between 2661.21 K and 6205.15 K, while the lowest color temperature in the zone with the lowest color temperature at the end of the color temperature cycle was between 2507.26 K and 6218.61 K. As shown in Table 3, the monthly extracted results were applied as thresholds to select the lowest color temperature candidates. Through STEP 1 and STEP 2, the lowest color temperature candidate was selected if it fell under the monthly expected lowest point occurrence zone and was included in the lowest color temperature threshold. In STEP 3, the lowest color temperature value was selected if it was the lowest even when compared with the 10 color temperature measurement values entered later. In addition, this process was equally applied to the search for the lowest color temperature around sunset.

After the lowest color temperature after sunrise was selected, a real-time natural light color temperature cycle was generated through the deep learning model TadGAN. TadGAN has the advantage of imitating and generating data with a pattern similar to the original as much as possible for time series data input to an unsupervised learning model [23]. Therefore, it could generate a periodic pattern for a lighting service for the irregular color temperature sequence of a specific time interval which was input in real-time. First, learning based on the dataset built in Section 2 was performed to generate a color temperature cycle by inputting measured color temperature in real-time. At this point, the learning environment was constructed using the Orion-ml library in the python environment, considering the previous study’s results [18]. The structure and detailed setting items of the TadGAN model were fixed as default values in the library. The learning was executed 100 times, the input data format was (100, 1), the batch size was 64, and the optimization algorithm was set to Adam [18].

**Figure 5:**
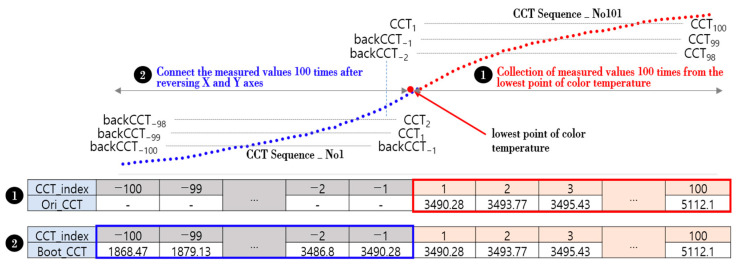
Boot data set creation process.

A deep learning model (TadGAN) to which the learning data of the color temperature cycle is applied can generate the first output (color temperature cycle) only when the time series data of a particular sequence is input. Therefore, when the existing TadGAN is applied as it is, a waiting time is required while the time series sequence data set in the model matches the processing unit window size, and a certain number of time series sequences are accumulated and input. However, the color temperature cycle would be missing since the generated color temperature cannot give an output during the waiting time. To solve this problem, the concept of booting and ending data sets was proposed and applied in this study. TadGAN needs boot data for the initial start, and if sufficient boot data is provided, performance stability can be improved. Accordingly, in the boot stage, which started the generation of the color temperature cycle, N time series sequence data before and after each of the time series sequence data were secured based on the occurrence time of the lowest point of the color temperature. In this case, N was set to 100, which was the sliding window size of the TadGAN learning data. Through this process, a boot data set consisting of 200 color temperature time series data was created, and the process is shown in Figure 5.

As shown in Figure 5, when the lowest point of the color temperature was determined after sunrise, 100 measured color temperature values were accumulated and collected, as shown in ①. Afterward, the process of ② was performed in which the accumulated measured color temperature value was reversely moved up and down, left and right, and the boot data set was constructed by connecting them. The formula for creating the boot data set (BootCCT) is shown in (1).

(1) $$CCT_{cycle}=\left(CCT_{init},\;CCT_{1},\;CCT_{2},\;\ldots \ldots \;CCT_{N}\right),\;N\;is\;time\left(CCT_{init}=CCT_{0}\right)\;BootCCT=\left\{backCCT_{-100},\;\ldots \ldots ,\;backCCT_{-1},\;CCT_{1},\;\ldots \ldots ,\;CCT_{100}\right\}\;backCCT_{-i}=\{\begin{array}{c} CCT_{-i}\;,\;i=1\\ backCCT_{-i+1}-\left|CCT_{-i}-CCT_{i-1}\right|\;,1<i\leq 100 \end{array}\}$$

After the boot data set was created, 100 color temperature time series sequences (CCT_Sequence_No1–CCT_Sequence_No100) were constructed and sequentially input to TadGAN. After that, by recursively applying the generator of TadGAN, it gave an output of the color temperature (TadCCT_i) each time in real-time [19].

(2) $$TadCCT_{cycle}=\left(TadCCT_{0},\;TadCCT_{1},\;\ldots \ldots \;TadCCT_{i}\right),\;i\;is\;time$$

The same concept was applied to the lowest point of the color temperature before sunrise, which corresponded to the end of the color temperature cycle; to construct the Ending Dataset and the time series sequence of the actual natural light color temperature, including the booting and ending datasets. The time series sequence of the actual natural light color temperature was input into TadGAN to acquire an output of the generated color temperature by the hour. The output color temperature was connected by time to generate a real-time daily color temperature cycle (TadCCT_cycle) for natural light reproduction.

## 4. Experiments and Analysis

**Figure 6:**
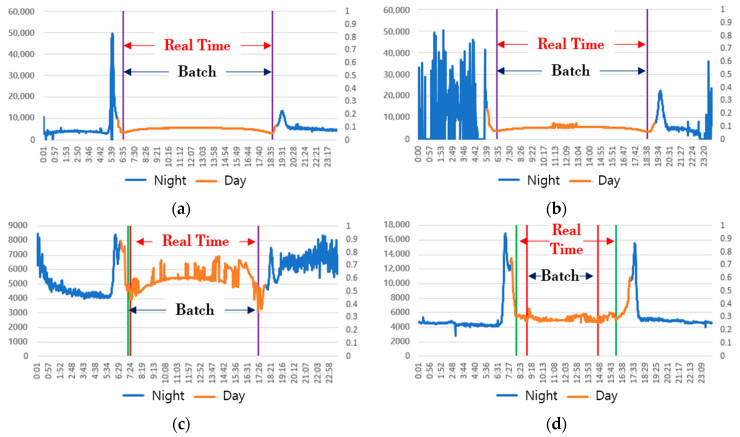
Search results of the lowest color temperature point and the color temperature cycle zone. (**a**) Irregular color temperature inclusion ratio of 0% (8 April 2020); (**b**) Irregular color temperature inclusion ratio of 10% (27 April 2020); (**c**) Irregular color temperature inclusion ratio of 30% (15 October 2019); (**d**) Irregular color temperature inclusion ratio of 50% (11 December 2020).

Experiments were conducted to confirm the lowest point search function of color temperature, an essential function for the generation of the real-time natural light color temperature cycle, and the generation performance of the natural light color temperature cycle. First, it was checked whether it was possible to select a zone of the color temperature cycle in real-time for the daily color temperature of natural light input in real-time by searching the start and end points of the color temperature cycle. For that, the proposed lowest point search function of the color temperature was applied to days with 0, 10, 30, and 50% ratios of color temperature anomalies and the results are shown in Figure 6. In the figure, “Real Time” represents the result of applying the proposed method, and “Batch” shows the lowest point of color temperature found by the researcher just before and immediately after sunrise and sunset for the daily color temperature cycle.

On a sunny day with a constant color temperature cycle in Figure 6a,b, the point and zone where the real-time search and the batch search were almost identical were selected as the lowest point and cycle zone of the color temperature. It was confirmed that the lowest point of the color temperature, which was almost similar to the batch search, was searched even on days with an anomaly ratio of the color temperature of 30% due to the influence of weather or atmospheric conditions in Figure 6c. However, on days when the rate of inclusion of anomalies was high, at 50%, and no parabolic color temperature cycle was observed, as in Figure 6d, a search was executed on a broader zone different from the batch search results.

On a sunny day with a constant color temperature cycle in Figure 6a,b, the point and zone where the real-time search and the batch search were almost identical were selected as the color temperature’s lowest point and cycle zone. It was confirmed that the lowest point of the color temperature, which was almost similar to the batch search, was searched even on days with an anomaly ratio of the color temperature of 30% due to the influence of weather or atmospheric conditions in Figure 6c. However, on days when the rate of inclusion of anomalies was high, at 50%, and no parabolic color temperature cycle was observed, as in Figure 6d, a search was executed on a broader zone different from the batch search results. As in Figure 6a–c, even if an anomaly of color temperature was included, the search for the lowest point of the color temperature and the selection of the color temperature cycle zone were almost accurate when a parabolic distribution appeared. In Figure 6d, the same color temperature cycle could not be specified due to natural light color temperature distribution characteristics on that day. In the batch search, only the most uniform color temperature cycle was judged as the zone with natural light. Nonetheless, in the real-time search, the longest natural light provided to the ground was judged as the color temperature cycle zone, even if the color temperature pattern was not uniform. Therefore, it was confirmed that it could be applied to lighting technology to provide natural light characteristics.

**Figure 7:**
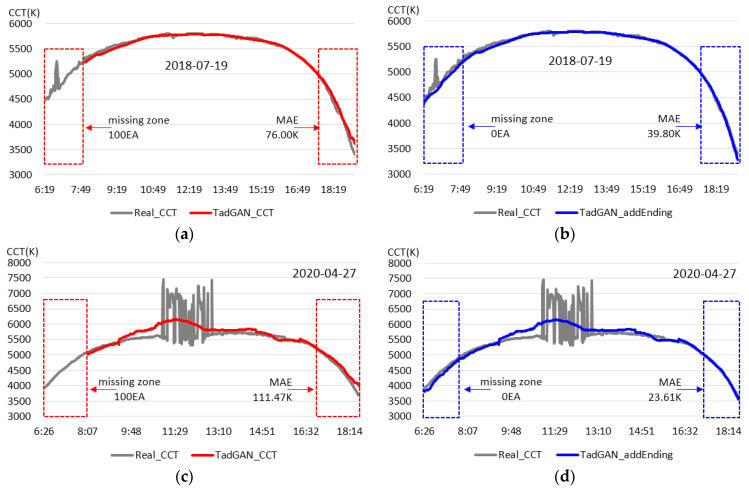
Real-time daily color temperature cycle generation result (irregular color temperature inclusion within 10%). (**a**) Irregular color temperature inclusion ratio of 2% (the conventional method); (**b**) Irregular color temperature inclusion ratio of 2% (the proposed method); (**c**) Irregular color temperature inclusion ratio of 10% (the conventional method); (**d**) Irregular color temperature inclusion ratio of 10% (the proposed method).

After the search for the lowest point of the color temperature, an experiment was conducted to confirm the generation performance of the real-time natural light color temperature cycle after applying the boot data set. TadGAN was applied assuming real-time input of color temperature cycles for clear and cloudy days selected by considering the inclusion ratio of irregular color temperatures. In this case, performance according to the application of the boot data set was evaluated based on the proposed method to which the boot data set was applied and the conventional method to which the boot data set was not applied. The experiment confirmed that a color temperature cycle similar to the original could be generated on days with a uniform color temperature cycle (irregular color temperature inclusion within 10%) even if irregular color temperatures were included. The experimental results are presented in Figure 7.

**Figure 8:**
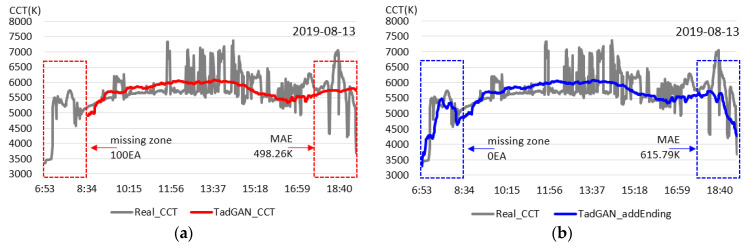
Results of generation of real-time daily color temperature cycle (irregular color temperature inclusion at 40%). (**a**) The conventional method, (**b**) the proposed method.

In the existing TadGAN, a color temperature cycle was not generated until the color temperature sequence data were collected after sunrise for TadGAN execution, as shown in Figure 7a,c. There was a common missing zone in which no color temperature cycle was generated for 100-time intervals. Around sunset, the color temperature cycle was generated differently from the original (measured) color temperature cycle pattern. However, as shown in Figure 7b,d, the proposed method gave the output of the color temperature cycle output from the start of the color temperature cycle generation and by resolving the problem of missing the color temperature cycle occurring after sunrise. Moreover, even in the section where the color temperature cycle ended, a color temperature cycle was generated in real-time, similar to the original color temperature pattern. When the mean absolute error (MAE) in the corresponding zone was compared, the MAEs for each day with irregular color temperature inclusion ratios of 2% and 10% were 76.00 K and 116.47 K, respectively, for the conventional method and 39.80 K and 23.61 K for the proposed method, confirming that the proposed method generated a color temperature cycle which represented the actual color temperature better. The experimental results, which checked the possibility of generating a real-time color temperature cycle on days with highly irregular color temperature cycles, are shown in Figure 8.

Since the measured color temperature for the day had a significantly irregular distribution, it was not easy to apply it as a standard for the color temperature cycle for lighting services. When the existing TadGAN was applied, it became possible to create a cycle even though there were missing zones. However, the color temperature cycle at sunset continued to maintain a high level, which was different from the pattern of the color temperature cycle on sunny days, as shown in Figure 6a,b, and which seemed unreasonable to apply to lighting services. Therefore, it did not seem easy to implement in the lighting service. Nonetheless, as shown in Figure 6b or the proposed method, a pattern was formed in the direction of a low color temperature cycle in the corresponding zone even in the irregular change of color temperature, making it possible to create a color temperature cycle for lighting service on the target day.

## 5. Conclusions

In lighting and architecture, technologies that exhibit natural light characteristics unique to each region in real-time to provide beneficial light to humans are being studied. Since there is difficulty in generating a color temperature cycle for color temperatures showing various irregular change patterns, there are cases where lighting service is practically implemented. This study proposes a method of generating a real-time natural light color temperature cycle to realize a circadian lighting service. After detecting the zone of the color temperature cycle for the constantly changing input color temperature, the boot and ending datasets were applied, and the daily color temperature cycle was generated through the deep learning (TadGAN) technique. First, the characteristics of the daily and monthly color temperature cycle of natural light were analyzed based on the natural light characteristic database that was measured and collected using the spectroradiometer for more than one year. For the circadian light service, the start and end of the color temperature cycle had to be determined at sunrise and sunset when the color temperature changes rapidly and on cloudy days when the color temperature cycle pattern was unclear. Therefore, the information on sunrise and sunset times provided by the astronomical institution and the measurement time of color temperature were compared. The data were checked to see if these fell under after sunrise and before sunset and under monthly color temperature cycle distribution. The lowest color temperature was selected when it was the lowest by comparing the 10 color temperature values entered after that. The daily color temperature cycle was defined as the interval between the lowest value of color temperature after sunrise and before sunset. A data set was then constructed by extracting the daily color temperature of clear days with an irregular color temperature within 15%. Boot data that connected 100-time series color temperature input values symmetrically inverted horizontally and vertically at the beginning and end of the color temperature cycle were applied to resolve the problem of performance degradation at the beginning and end of the color temperature cycle when TadGAN was applied, which was confirmed in a previous study. This process created a more accurate color temperature cycle at the beginning and end of the daily color temperature cycle, significantly influencing human health. After applying the daily lowest point search technique and booting data set, TadGAN was found to generate a real-time natural light color temperature cycle, and the applicability of the proposed method was confirmed through experiments. The results showed that the color temperature cycle search method detected a color temperature cycle zone almost identical to the batch processing method collectively designated by the user. Moreover, the generation performance of the real-time color temperature cycle was compared for several days with different anomaly ratios. The proposed method solved the problem of the missing color temperature cycle, which occurred to construct the input sequence of TadGAN at the beginning of the color temperature cycle. Additionally, on days with a 2% and 10% irregular color temperature inclusion ratio, the proposed method showed MAEs of 39.80 and 23.61 K and 76.00 and 116.47 K for the conventional method, respectively. The proposed method created a real-time color temperature cycle more consistent with the original. Furthermore, the real-time color temperature cycle for the lighting service could be generated even when it was challenging to generate a periodic pattern with an irregular color temperature inclusion ratio of 40%.

In the future, additional research is required for commercialization that collects and analyzes natural light characteristics by linking small sensing devices other than spectroradiometers and applying time series sequences of various time intervals. Likewise, it is necessary to study the advancement and commercialization of the proposed method so that the generation of a real-time color temperature cycle is possible even for the input of changing color temperature according to various weather or atmospheric environments. Based on these studies, efforts to develop a real-time circadian lighting system may be continued.

## Figures and Tables

**Table 1:** The ratio of anomaly inclusion in the daily measured natural light color temperature.

Category	The Ratio of Inclusion of Irregular Color Temperature (%)														
	0–5	5–10	10–15	15–20	20–25	25–30	30–35	35–40	40–45	45–50	50–55	55–60	60–65	65–70	70–75
Days (Day)	71	47	30	30	36	28	17	13	8	9	3	2	2	1	2
Cumulative percentage (%)	23.8	35.6	49.7	59.7	71.8	81.2	86.9	91.2	94.0	97.0	98.0	98.7	99.0	99.3	100

**Table 2:** The process of finding the lowest point of color temperature.

STEP 0		STEP 1		STEP 2		STEP 3
Collection of sunrise and sunset time information	▶	Comparison monthly where the lowest zones were expected	▶	Selection of threshold based on the lowest color temperature candidate	▶	Determination of the lowest color temperature through comparison by zone

**Table 3:** Occurrence of the lowest monthly color temperature.

	Month	Winter		Spring			Summer			Autumn			Winter
Item		1	2	3	4	5	6	7	8	9	10	11	12
Duration from sunrise to the lowest point (relative time %)		75 (12.67)	74 (11.48)	86 (12.10)	73 (9.32)	88 (10.59)	90 (10.29)	127 (14.85)	73(9.10)	72(9.90)	59(8.89)	68 (11.34)	67 (11.67)
The lowest color temperature zone in the morning		2989K~ 5949K	3183K~ 5759K	3320K~ 6063K	3273K~ 5963K	3319K~ 6198K	2981K~ 6176K	3222K~ 6205K	2895K~ 5580K	3256K~ 6069K	3153K~ 6045K	3086K~ 6024K	2661K~ 5479K
Duration from the lowest point to sunset (relative time %)		83 (13.97)	78 (12.11)	77 (10.91)	68 (8.67)	87 (10.48)	88 (10.14)	103 (12.01)	58 (7.21)	76 (10.38)	50 (7.50)	65 (10.80)	76 (13.25)
The lowest point color temperature zone is in the afternoon		2944K~ 6027K	2949K~ 5981K	3065K~ 6123K	3208K~ 6096K	2796K~ 6043K	2988K~ 6120K	2976K~ 6218K	3204K~ 5998K	2690K~ 6135K	2901K~ 6047K	2841K~ 6058K	2507K~ 5550K

## Data Availability

Not applicable.

## References

[1] Knoop M., Stefani O., Bueno B., Matusiak B., Hobday R., Wirz-Justice A., Martiny K., Kantermann T., Appelt S. (2020). Daylight: What makes the difference?. Light. Res. Technol..

[2] Ellis E.V., Gonzalez E.W., Kratzer D.A., McEachron D.L., Yeutter G. (2013). Auto-tuning daylight with LEDs: Sustainable lighting for health and wellbeing. ARCC Conf. Repos..

[3] Wahl S., Engelhardt M., Schaupp P., Lappe C., Ivanov I.V. (2019). The inner clock—Blue light sets the human rhythm. J. Biophotonics.

[4] Lockley S.W., Brainard G.C., Czeisler C.A. (2003). High sensitivity of the human circadian melatonin rhythm to resetting by short wavelength light. J. Clin. Endocrinol. Metab..

[5] Gaston K.J., Duffy J.P., Gaston S., Bennie J., Davies T.W. (2014). Human alteration of natural light cycles: Causes and ecological consequences. Oecologia.

[6] Zhang R., Campanella C., Aristizabal S., Jamrozik A., Zhao J., Porter P., Ly S., Bauer B.A. (2020). Impacts of dynamic LED lighting on the well-being and experience of office occupants. Int. J. Environ. Res. Public Health.

[7] Mangkuto R.A., Aries M.B.C., Loenen E.V., Hensen J.L.M. (2014). Simulation of virtual natural lighting solutions with a simplified view. Light. Res. Technol..

[8] Su Z., Zhao B., Gong Z., Peng Y., Bai F., Zheng H., Joo S.W. (2021). Color-tunable white LEDs with single chip realized through phosphor pattern and thermal-modulating optical film. Micromachines.

[9] “Dyson lightcycle” Dyson. https://www.dyson.com/lighting/task-lighting/dyson-lightcycle/dyson-lightcycle-overview.

[10] Blazejczyk K., Morita T., Towatari T., Blazejczyk A., Wieczorek J. (2014). Seasonal and Regional Differences in Lighting Conditions and their Influence on Melatonin Secretion. Quaest. Geogr..

[11] Konis K. (2013). Evaluating daylighting effectiveness and occupant visual comfort in a side-lit open-plan office building in San Francisco, California. Build. Environ..

[12] Oh S.T., Jeon G.W., Lim J.H. (2020). Method of Calculating Short-Wavelength-Ratio-Based Color Temperature Supporting the Measurement of Real-Time Natural Light Characteristics through RGB Sensor. Sensors.

[13] Wirz-Justice A., Skene D.J., Münch M. (2021). The relevance of daylight for humans. Biochem. Pharmacol..

[14] Ming A., Rowell I., Lewin S., Rouse R., Aubry T., Boland E. (2021). Key messages from the IPCC AR6 climate science report. Camb. Open Engag..

[15] Dixon E.R. (1978). Spectral distribution of Australian daylight. JOSA.

[16] Jeon G.W., Oh S.T., Lim J.H. (2021). Algorithm for Judging Anomalies Using Sliding Window to Reproduce the Color Temperature Cycle of Natural Light. J. Korea Multimed. Soc..

[17] Kim K.M., Kim Y.W., Oh S.T., Lim J.H. (2020). Development of a natural light reproduction system for maintaining the circadian rhythm. Indoor Built Environ..

[18] Geiger A., Liu D., Alnegheimish S., Cuesta-Infante A., Veeramachaneni K. TadGAN: Time series anomaly detection using generative adversarial networks. Proceedings of the 2020 IEEE International Conference on Big Data (Big Data).

[19] Lee S.H., Kim Y.S. (2022). A Pre-processing Process Using TadGAN-based Time-series Anomaly Detection. J. Korean Soc. Qual. Manag..

[20] Oh S.T., Ga D.H., Lim J.H. (2022). TadGAN-Based Daily Color Temperature Cycle Generation Corresponding to Irregular Changes of Natural Light. Sensors.

[21] Hu X., Xu T., Zhou L., Wang Q., Arıcı M., Li D. (2019). Comparison of transmittance and reflection methods for solving optical constants of optical glass. Optik.

[22] “Quartz Disk” Hanjin Quartz. https://www.hjquartz.com/home/mobile_img_view/TUk4MDc3.

[23] Goodfellow I., Pouget-Abadie J., Mirza M., Xu B., Warde-Farley D., Ozair S., Courvill A., Bengio Y. (2020). Generative adversarial networks. Generative adversarial networks. Commun. ACM.

